# Antioxidant effects of calligonum extract on ovarian tissue of PCO model: An experimental study

**Published:** 2018-10

**Authors:** Fatemeh Tahmasebi, Mansoureh Movahedin, Zohreh Mazaheri

**Affiliations:** *Department of Anatomy, Faculty of Medical Sciences, Tarbiat Modares University, Tehran, Iran.*

**Keywords:** Polycystic ovary syndrome, Antioxidant, In vitro fertilization, Mouse, Calligonum

## Abstract

**Background:**

Polycystic ovary syndrome (PCO) is one of the most common reasons for infertility. Calligonum as a plant possess some of the important antioxidants that can decrease oxidative stress.

**Objective:**

The effects of treatment with Calligonum as an antioxidant on ovary tissue of a PCO mouse model.

**Materials and Methods:**

Thirty female NMRI mice were divided into three groups (n=10/each): control, PCO, and Calligonum. We induced PCO model with single dose of Estradiol valerate (40 mg/kg). Then Calligonum (20 mg/kg) was intraperitoneally injected weekly for two months. The level of oxidative stress and total antioxidant capacity was assessed in the ovarian tissue by flow cytometry and fluorescence recovery after photobleaching, respectively, and the histological study was conducted by the morphometric method and embryo development with in vitro fertilization.

**Results:**

The obtained results showed that estradiol valerate was able to increase oxidative stress within the ovary and causes ovarian cysts after two months. The cyst formation was decreased in Calligonum group compared to PCO group (p=0.001). The percentage of pre-antral and antral follicles significantly decreased in Calligonum group compared to PCO group (p=0.001). The oxidative stress decreased in Calligonum group significantly compared to PCO group (p=0.001). Calligonum can significantly increase the total antioxidant capacity of ovarian tissue (p=0.001) as well as the percentage of in vitro fertilization compared to the PCO group.

**Conclusion:**

Calligonum could decrease ovary cyst in PCO model, and improve in vitro fertilization rate. Also, Calligonum extract as an antioxidant could decrease oxidative stress in PCO model.

## Introduction

Infertility is a major problem in modern societies and polycystic ovary syndrome (PCO) impress 5-20% of women of pregnancy age worldwide and is among the causes of infertility in women and it’s a common disorder that (1). PCO disorders include lack of ovulation, ovarian filled clear cysts, hyperandrogenism, and metabolic disorders such as obesity (2). 

A suitable animal model can be valuable in studying its pathogenesis and help in making a more effective diagnosis and choosing appropriate treatments. The balance in reactive oxygen species (ROS) plays an important physiological role in several processes of the female reproductive system, including oocyte maturation, ovulation, fertilization, and endometrial loss (3). Anti-oxidative enzymes are include catalase, superoxide dismutase and glutathione peroxidase that they have protective mechanisms against the oxidative stress effects (4). These factors contribute to the balanced production of ROS by cells in the body (5). 

Several plants such as Allium cepa, Pimpinella anisum, and Calligonum contain antioxidant compounds (6). Calligonum is a member of Polygonaceae family and is a drought-tolerant shrub adapted to conditions of water scarcity that grows in the deserts and sandy soils (7). Several antioxidative compounds have been known from the Calligonum extract, consist of dehydrodicatechin A, catechin, kaempferol-3-O-rhamnopyranoside, isoquercetin (quercetin-3-O-glucopyranoside), quercetin (quercetin-3-O-rhamnopyranoside), and kaempferol-3-O-glucuronide (6, 8). This extract could be an antioxidant component and use for ROS reduction (9). 

Quercetin is a critical element of Calligunum that modulates ovarian functions and it can control cell steroidogenic activity (10). The quercetin inhibit the Toll-like receptor/NF-κB pathway and reduce the inflammatory effect in the ovarian tissue of the PCO model (11). Catechin is another key element of Calligonum that can reduce LH serum level, body and ovarian weight, insulin resistance index in the treatment groups related to PCO. The studies confirmed significant changes in the theca layer thickness and number of follicles. These changes demonstrated improvement in the PCOS symptoms which is due to antioxidants effects on ROS pathways (12). 

In this study, we assessed the protective effects of Calligonum comosum extract on oxidative stress in a mouse model of the polycystic ovary. The aim of this study was that Calligonum with antioxidant properties can improve PCO symptoms and it is useful for women health.

## Materials and methods

Thirty adult female NMRI mice (25 gr) with the aged of 8 wk obtained from Pasteur institute (Iran). The animals were housed under a 12 hr light/dark cycle in a room with controlled temperature (23±2^o^C) and free access to food and water. The mice were randomly divided into three groups (n=10/each): I: Control, II: PCO: Received a single intramuscular injection of estradiol valerate (EV) (40 mg/kg) according to previous studies (13, 14), III: Calligonum: A single injection of EV followed by 20 mg/kg intraperitoneal Calligonum extract per week (11).

In this study, the Calligonum extract 20 mg/kg was intraperitoneally injected one day after EV 40 mg/kg injection weekly for two months. EV was used in this study to create a polycystic ovarian model because it is readily available, cheap and does not require multiple injections. The model was developed according to a previous study (13). After 8 wk of treatment, all the mice were sacrificed with cervical vertebrae displacement. The histological preparation was done and the ROS level, total antioxidant capacity, and fertilization were assessed in all the three groups.

**Measurement of body weight**

Within eight wk of a single EV injection, the weight of mice was measured weekly. After each weighting, 20 mg/kg of Calligonum extract was injected into the mice according to the amount of gained weight.

**Histological and morphometry methods**

Tissue sections were prepared according to a previous study (13). After Haemotoxylin and Eosin staining, the follicle count slides were prepared and evaluated by light microscope. Ovary sections were examined under ×3.2 magnification for corpus luteum, as well as ×14 and ×40 for follicles. A number of ovarian tissue sections were removed to a ratio of 1-4. The types of follicles and corpus luteum were counted spirally clockwise to the medulla (15). The number of corpus luteum and ovarian follicles were compared in the three groups, including control, PCO, and Calligonum. In addition to a variety of follicles, a number of cysts were in the ovary. In each section, to prevent the repetition of a follicle count, only follicles with distinct nucleoli were counted.

**The measurement of oxidative stress in ovarian tissue**

Flow cytometry was used to assess ROS level in ovarian tissue (16). In this method, fluorescent probes are used for the detection of intracellular ROS. Oxidation of Dichloro-dihydro-fluorescein diacetate (DCFH-DA) (Sigma, Germany) by ROS produced in the cells increases their fluorescence and can be used for measurement of hydrogen peroxide. Thus, the ovarian tissue of mice was removed after 8 wk of treatment. The tissue was completely lysed by a homogenizer and was then centrifuged at 2500 rpm at 4^o^C for 5 min. The medium on plaques was exchanged with PBS and twice centrifuged at 1000 rpm at 4^o^C for 3 min. In the dark, 10 µl of 20 µM, DCFH-DA was added to the tissue and was slowly pipetted. Then, it was incubated for 45 min at 37^o^C. After a period of incubation in the dark, 900 µl of PBS was added and centrifuged at 2500 rpm in 4^o^C for 5 min and was analyzed by flow cytometry.

**Measurement of total antioxidant capacity in ovarian tissue by Fluorescence recovery after photobleaching (FRAP) test**

The water-soluble antioxidants in the sample cause reduction of the Fe^3+-^ 2,4,6-Tris(2-pyridyl)-s-triazine complex to Fe^2+-^ 2,4,6-Tris(2-pyridyl)-s-triazine, which has a blue color in the acidic environment with maximum light absorption in 593 nm wavelength. Standard solutions were prepared in 125, 250, 500, 1000 µM concentrations. 1.5 ml FRAP solution was added to 50 µl sample and was completely vortexed. After 10 min of incubation at room temperature (37^o^C), the absorbance of all samples was read in 593 nm in front of the blank (zero concentration standard) and FRAP of unknown samples was calculated based on a curve (17). 

**In vitro fertilization (IVF)**

The mice were sacrificed by cervical vertebrae dislocation, their abdominal cavity was opened and epididymis was separated and put into1-ml drops [Human tubal fluid (HTF) without 4-(2-hydroxyethyl)-1-piperazineethanesulfonic acid (HEPES) containing 15 mg Bovine serum albumin (BSA) Then, cross-sections were made by a fine needle and incubated at 37^o^C, 6.2% CO_2_, and 100% moisture for 10 min to drive the sperms out of the tube. Active and healthy sperms that were gathered on the edge of drops with swim up were added to droplets containing the MII eggs prepared for IVF.

To stimulate the ovulation, 10 IU of pregnant mare serum gonadotropin (PMSG, Sigma, Germany) was injected followed by 10 IU Human Chorionic Gonadotropin (hCG, Sigma-Aldrich, Germany) (11) after 48 hr. 18-20 hr after injection of HCG, the mice were sacrificed, their oviduct was removed and MII oocytes were put in 100 µl droplets under oil. The culture droplets contained HTF with 5 mg/ml HEPES+BSA. MII oocytes were removed when the ampule of oviduct was ruptured. Then, MII oocytes were washed in one droplet and transferred to another plate containing the droplets for IVF (including HTF without HEPES+BSA up to 15 mg/ml).

A volume of sperm (containing 150,000 spermatozoids) was added to 50 μl droplets containing MII oocytes for IVF. IVF was considered to be terminated after 4-6 hrs. Then, the zygotes were transferred to drops for embryo culture (global+BSA 5 mg/ml). The cells were cultured for one day and fertilization rates were reported (18).

**Ethical consideration**

All procedures on these animals were approved by the Medical Ethics Committee of Tarbiat Modares University (52/6708). All research and animal maintenance procedures were done according to international guidelines on the use of laboratory animals. It was attempted to minimize the number of animals and their suffering.

**Statistical analysis**

The results of this study were analyzed by SPSS software (Statistical Package for the Social Sciences, version 20.0, SPSS Inc, Chicago, Illinois, USA), one-way ANOVA and Tukey post hoc tests. Results of ROS, body weight, morphometry, and total antioxidant capacity (TAC) in experimental groups were analyzed by One Way ANOVA test. The in vitro fertilization was assessed by Chi-Square test. A correlation test (Bivariate) was performed for mean concentration of water-soluble antioxidants and ROS average. The data of all stages of this study was calculated by mean±standard deviation (SD). p≤0.001 was considered as the significance value.

## Results

**Assessment of animal body weight**

**Figure 1:**
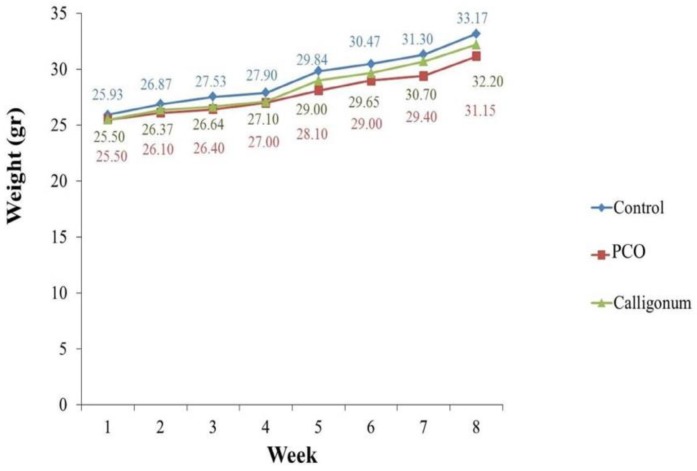
The mean changes in body weight of mice weekly for 8 wk. There was not significantly different between the three groups. Data are means ± SD

Within 8 wk of PCO model induction, body weights were measured in control, PCO, and Calligonum groups. The results of this study showed that the average weight of mice in all groups was significantly increased (p=0.001), but average weight gain in the two groups was less than the control group. The body weight was not significantly (p=0.1) different between the three groups (Figure 1). 

**Assessment of ovarian tissue histopathology after administration of Calligonum extract**

**Figure 2:**
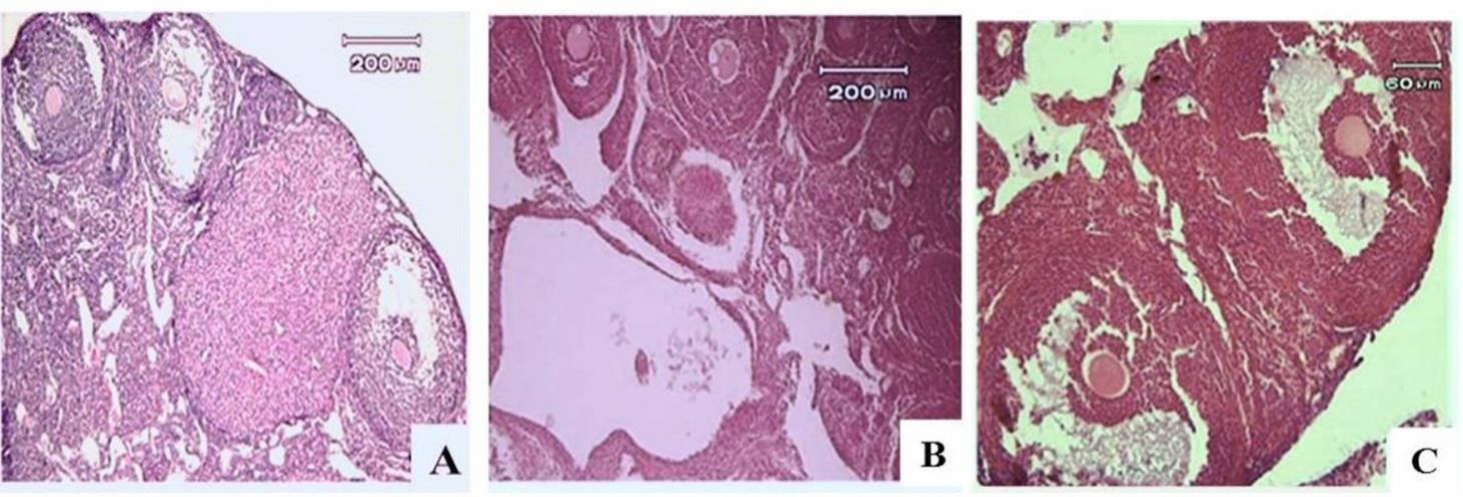
Histological analysis of ovarian tissue in control group (A), PCO group (B) and Calligonum group (C). A lot of cysts were seen in the tissue of the ovary in PCO group compared to the other groups. H & E staining (Magnification ×200).

After two months, the results of the study expressed that ovarian sections in Calligonum group in contrast to PCO group, showed no large cysts in the ovarian cortex and the number of small cysts in medulla was decreased. In addition, the growth of pre-antral follicles was ceased in PCO, but antral follicles were observed in the Calligonum group. Also histological analysis of ovarian sections in PCO showed the accumulation of cystic follicles, preantral follicles, lack of ovulation, as well as corpus luteum. While in control group, the presence of a large number of corpus luteum was an indicator of the normal sexual cycle (Figure 2). 

**Morphometric study of ovarian tissue after Calligonum extract treatment**

**Figure 3:**
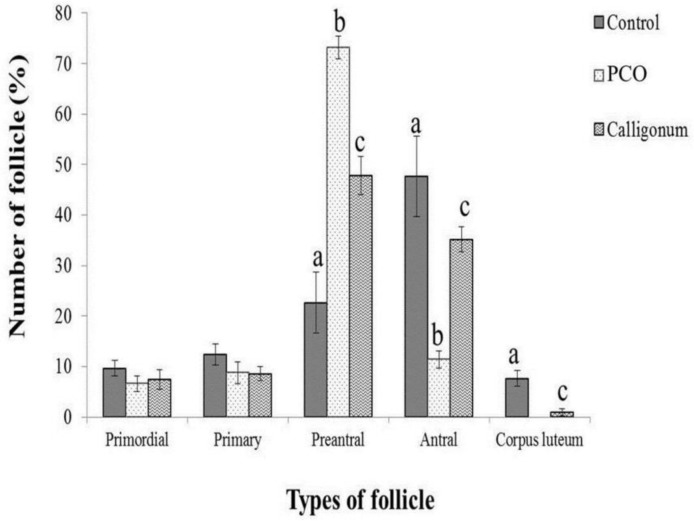
Morphometric study of ovarian tissue after the administration of Calligonum to PCO mice compared to PCO and control groups. Data are means ± SD, n=5. a, b, c: significant differences (p=0.001) compared to other groups

According to the results, the percentage of primordial and primary follicles between the groups was not significantly different. The percentage of pre-antral and antral follicles among all the three groups had a significant difference (p=0.001). The highest percentage of antral follicles was observed in the control group and the lowest in PCO group (Figure 3).

**Evaluation of oxidative stress in the ovarian tissue after administration of Calligonum extract**

**Figure 4:**
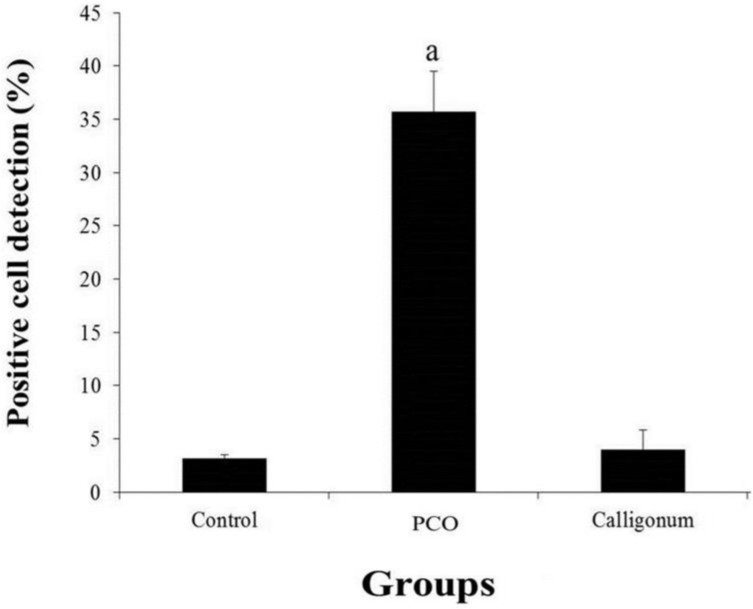
Assessment ROS levels in ovarian tissue in the three groups. Data represent means ± SD, n=5. a: significant differences (p=0.001) compared to other groups

The results showed that the lowest average reflectance of fluorescent DCF (ROS level in cells of ovarian tissue) was observed in the control group. In addition, the highest level of ROS was observed in PCO group. The level of ROS in PCO was significantly different (p=0.001) compared to other groups (Figure 4).

**Evaluation of the total antioxidant capacity of ovarian tissue**

According to table I, the results showed the lowest concentration of antioxidant in PCO group and the highest in Calligonum group. The results showed significant differences (p=0.001) between groups. In this test, the correlation between the two variables showed a mean inverse relationship in control group between average fluorescence reflectance with a concentration of soluble antioxidants and a strong inverse relationship in PCO and Calligonum groups.

**Evaluation of in vitro fertilization after the administration of Calligonum**

Based on the results, the total number of eggs obtained from PCO group after ovulation induction was lesser than the other groups. According to table II, the lowest percentage of in vitro fertilization was observed in PCO, while the highest percentage was in the control group. Fertilization rate had a significant difference (p=0.001) in PCO group with the other groups (Table II). 

**Table I:** Assessment of ROS, TAC, and correlation between them

**Groups**	**ROS**	**Mean concentration antioxidant**	**Correlation between ROS and TAC**
Control	3.11± 0.35	338.37^c^± 20.21	(Mean inverse relationship)-0.588
PCO	35.63^a^± 3.81	148.87^b^ ±5.26	(Strong inverse relationship)- 0.795
Calligonum	3.94 ± 1.84	479.93^a^ ±29.62	(Strong inverse relationship)- 0.9

Data are means ± SD.

ROS: Reactive oxygen species

TAC: Total antioxidant capacity

PCO: Polycystic ovary

a, b, c : significant differences (p=0.001) compared to other groups

**Table II:** Evaluation of in vitro fertilization after the administration of Calligonum to PCO group compared to PCO, control

**Groups**	**MII (number)**	**Two cell number ** **(%)**
Control	128	88.9
PCO	56	50^a^
Calligonum	107	85.6

Data are means ± SD,

PCO: Polycystic ovary

a: significant differences (p=0.001) compared to other groups.

## Discussion

According to the results of this study, the average weight of mice was significantly increased over time during 8 wk of PCO model induction in all groups, but the mean of two groups was less than the control group. This weight loss was not statistically significant. Obesity and overweight are two symptoms of PCO (19). These results were similar to Stener-Victorin results (20). It can be concluded that EV will not affect the weight of mice 8 wk after injection. Studies within a period longer than 8 wk may indicate some effects on weight. 

The assessment of preventive effects of Calligonum on ovarian tissue showed that the injection of extract simultaneous with PCO leads to lack of formation of large cysts in cortex and reduced number of small cysts in the medullary region compared to PCO. In addition, abundant pre-antral follicles were observed in PCO group, but in Calligonum group, many antral follicles were growing from the pre-antral stage. Amini and colleagues in 2015 studied effects of Calligonum comosum on ovarian histology of PCO mouse model. They expressed that there was no significant effect of 20 mg/kg Calligonum on polycystic ovarian morphology mouse model that it was opposite to our study (21).

Morphometry was used to quantify the ovarian follicles in the ovarian tissue (22). The index of the cystic ovary in follicular growth was stopped and no corpus luteum was seen in it (23). Our morphometric study of ovarian sections in PCO group showed the accumulation of pre-antral follicles and lack of corpus luteum, which indicate lack of ovulation. A small number of corpus luteum was observed in Calligonum group. While a large number of corpus luteum were observed in control samples as a sign of normal sexual cycle. According to these findings, we can conclude that the injection of 20 mg/kg Calligonum extract to a mouse model of PCO can be prevented or ceased in the pre-antral stage for follicle and reduce the number of cysts.

Ghasemzadeh and colleagues in 2013 investigated the effects of an antioxidant extract of Allium Cepa in rats subject to PCO induction with EV. In PCO group treated with Allium Cepa extract, the number of apoptotic granulosa cells was significantly reduced compared to PCO without treatment which is similar to our study (24). 

ROS is one of the most important indicators for measuring antioxidant effects of plant extracts (25). Therefore, in this study, ROS was evaluated in different groups according to the hypothesis that antioxidants reduce the ROS level. The ROS level showed that the oxidative stress induced by PCO in ovarian tissue was much higher than the other groups. In addition, the level of ROS in Calligonum group was less than PCO. The results indicate that the plant extract with antioxidant properties reduced ROS induced by EV. 

Zhang and colleagues stated that sodium selenite indirectly acts as an antioxidant enzyme and can form a ferric salt, which is able to transport iron in the cell membrane and is essential for respiration and cell metabolism. This is a mechanism for beneficial effects of sodium selenite and provides cell growth and prevents iron accumulation, as well as the formation of hydroxyl radicals through Fenton reaction that is similar to our results for ROS reduction (26). 

The effect of 20 mg/kg dose of the Calligonum extract on antioxidant levels in ovarian tissue of PCO group showed the lowest concentration of antioxidant in PCO group and the highest in the group treated with plant extract. Jahan and colleagues in 2018 studied the therapeutic potentials of Quercetin in management of PCO. Their findings showed that quercetin is a powerful flavonoid that impress metabolic and endocrine system in PCOS. Also, it showed strong antioxidant potentials and recovered ovarian cysts, healthy follicles (27). The results of correlation between antioxidant levels and ROS showed a strong inverse relationship in PCO group so that EV increased ROS and decreased the antioxidants levels in ovarian tissue. In Calligonum group, there was a strong inverse relationship. In the control group, there was a mean inverse relationship. 

Based on the results, the total number of obtained eggs from PCO group was much less than the other groups after induction of ovulation in mice to perform IVF. The quality and structure of egg are important in fertilization process; low quality of eggs and loss of its ultrastructure is a common problem in infertile women with PCO (28). Results in this stage showed that the released MII oocytes were increased in the group treated with plant extract. Based on these results, the extract is likely to increase the growth of supportive granulosa cells for oocytes and the interaction between them led to the production of eggs with a high fertility rate. Qian and his colleagues in 2016 reported that H_2_O_2_ concentration was higher in fragmented embryos and unfertilized eggs. In fact, this study demonstrated that increased oxidative stress in ovary tissue produces eggs with low fertility and lower-quality embryos (29).

## Conclusion

Our data showed that the antioxidants in Calligonum extract (20 mg/kg) can reduce cysts in PCO model and eliminates free radicals and oxidative stress in ovary tissues. So, Calligonum extract can be used as an antioxidant component for preventing infertility.

## Note

***This article extracted from M.Sc. thesis. (Fatemeh Tahmasebi)***

## References

[1] Azziz R (2016). PCOS in 2015: New insights into the genetics of polycystic ovary syndrome. Nat Rev Endocrinol.

[2] Pinola P, Puukka K, Piltonen TT, Puurunen J, Vanky E, Sundström-Poromaa I (2017). Normo-and hyperandrogenic women with polycystic ovary syndrome exhibit an adverse metabolic profile through life. Fertil Steril.

[3] Lemos AJ, Peixoto CA, Teixeira ÁA, Luna RL, Rocha SW, Santos HM (2014). Effect of the combination of metformin hydrochloride and melatonin on oxidative stress before and during pregnancy, and biochemical and histopathological analysis of the livers of rats after treatment for polycystic ovary syndrome. Toxicol Appl Pharmacol.

[4] Sweazea KL, Johnston CS, Knurick J, Bliss CD (2017). Plant-based nutraceutical increases plasma catalase activity in healthy participants: A small double-blind, randomized, placebo-controlled, proof of concept trial. J Diet Suppl.

[5] Štajner D, Milić N, Čanadanović-Brunet J, Kapor A, Štajner M, Popović BM (2006). Exploring Allium species as a source of potential medicinal agents. Phytother Res.

[6] Badria FA, Ameen M, Akl MR (2007). Evaluation of cytotoxic compounds from Calligonum comosum L growing in Egypt. Z Naturforsch C.

[7] Ashour OM, Abdel-Naim AB, Abdallah HM, Nagy AA, Mohamadin AM, Abdel-Sattar EA (2012). Evaluation of the potential cardioprotective activity of some Saudi plants against doxorubicin toxicity. Z Naturforsch C.

[8] Abdel-Sattar EA, Mouneir SM, Asaad GF, Abdallah HM (2014). Protective effect of Calligonum comosum on haloperidol-induced oxidative stress in rat. Toxicol Ind Health.

[9] Askari Jahromi M, Movahedin M, Mazaheri Z, Amanlu M, Mowla SJ, Batooli H (2014). Evaluating the effects of Escanbil (Calligonum) extract on the expression level of Catsper gene variants and sperm motility in aging male mice. Iran J Reprod Med.

[10] Shah KN, Patel SS (2016). Phosphatidylinositide 3-kinase inhibition: A new potential target for the treatment of polycystic ovarian syndrome. Pharm Biol.

[11] Wang Z, Zhai D, Zhang D, Bai L, Yao R, Yu J (2017). Quercetin decreases insulin resistance in a polycystic ovary syndrome rat model by improving inflammatory microenvironment. Reprod Sci.

[12] Ghafurniyan H, Azarnia M, Nabiuni M, Karimzadeh L (2015). The effect of green tea extract on reproductive improvement in estradiol valerate-induced polycystic ovarian syndrome in rat. Iran J Pharm Res.

[13] Tahmasebi F, Movahedin M, Mazaheri Z (2015). [Poly cystic ovary model as an elevated oxidative stress factor. ] J Mazandaran Univ Med Sci.

[14] Peyghambari F, Amanpour S, Fayazi M, Haddadi M, Muhammadnejad S, Muhammadnejad A (2014). Expression of α4, αv, β1 and β3 integrins during the implantation window on blastocyst of a mouse model of polycystic ovarian syndromes. Iran J Reprod Med.

[15] Hasanzadeh S, Sadeghinejad J (2012). Histomorphometry of ovarian follicular growth and atresia in summer and winter seasons in Iranian river buffalo. Indian J Anim Sci.

[16] Silveira LR, Pereira-Da-Silva L, Juel C, Hellsten Y (2003). Formation of hydrogen peroxide and nitric oxide in rat skeletal muscle cells during contractions. Free Radic Biol Med.

[17] Zhang X, Zhang F, Gao XJ, Yong JJ, Zhang WH, Zhao JJ (2017). Effects of different drying methods on content of bioactive component and antioxidant activity in Lycium ruthenicum. Zhongguo Zhong Yao Za Zhi.

[18] Preaubert L, Courbiere B, Achard V, Tassistro V, Greco F, Orsiere T (2016). Cerium dioxide nanoparticles affect in vitro fertilization in mice. Nanotoxicology.

[19] Arentz S, Smith CA, Abbott J, Fahey P, Cheema BS, Bensoussan A (2017). Combined lifestyle and herbal medicine in overweight women with polycystic ovary syndrome (PCOS): A randomized controlled trial. Phytother Res.

[20] Stener-Victorin E, Kobayashi R, Watanabe O, Lundeberg T, Kurosawa M (2004). Effect of electro-acupuncture stimulation of different frequencies and intensities on ovarian blood flow in anaesthetized rats with steroid-induced polycystic ovaries. Reprod Biol Endocrinol.

[21] Amini L, Tehranian N, Movahedin M, Ramezani Tehrani F (2015). Effect of Calligonum Comosum on Ovarian Histology of Polycystic Ovary Mouse Model. J Med Plants.

[22] Urra J, Blohberger J, Tiszavari M, Mayerhofer A, Lara HE (2016). In vivo blockade of acetylcholinesterase increases intraovarian acetylcholine and enhances follicular development and fertility in the rat. Sci Rep.

[23] Duncan WC (2017). The corpus luteum and women’s health. The Life Cycle of the Corpus Luteum.

[24] Ghasemzadeh A, Farzadi L, Khaki A, Ahmadi SK (2013). Effect of Allium cepa seeds ethanolic extract on experimental polycystic ovary syndrome (PCOS) apoptosis induced by estradiol-valerate. Life Sci J.

[25] Poole KM, Nelson CE, Joshi RV, Martin JR, Gupta MK, Haws SC (2015). ROS-responsive microspheres for on demand antioxidant therapy in a model of diabetic peripheral arterial disease. Biomaterials.

[26] Zhang J, Robinson D, Salmon P (2006). A novel function for selenium in biological system: selenite as a highly effective iron carrier for Chinese hamster ovary cell growth and monoclonal antibody production. Biotechnol Bioeng.

[27] Jahan S, Abid A, Khalid S, Afsar T, Qurat-Ul-Ain, Shaheen G (2018). Therapeutic potentials of Quercetin in management of polycystic ovarian syndrome using Letrozole induced rat model: a histological and a biochemical study. J Ovarian Res.

[28] Jing L, Wei C, Wei S, Ji W (2014). Effect of electroacupuncture on egg quality and tumor necrosis factor-α of patients with polycystic ovarian syndrome. World J Acupuncture-Moxibustion.

[29] Qian D, Li Z, Zhang Y, Huang Y, Wu Q, Ru G (2016). Response of mouse zygotes treated with mild hydrogen peroxide as a model to reveal novel mechanisms of oxidative stress-induced injury in early embryos. Oxid Med Cell Longev.

